# A comprehensive approach to optimizing malaria prevention in pregnant women: evaluating the efficacy, cost-effectiveness, and resistance of IPTp-SP and IPTp-DP

**DOI:** 10.1080/16549716.2023.2231257

**Published:** 2023-07-17

**Authors:** Sarah-Leah Eisenberg, Adam E. Krieger

**Affiliations:** a Mailman School of Public Health, Columbia University, New York, NY, USA; b Faculty of Medicine, Technion, Haifa, Israel; c Davis School of Gerontology, University of Southern California, Los Angeles, CA, USA

**Keywords:** Malaria, pregnancy, healthcare access, patient compliance, health education

## Abstract

Malaria during pregnancy is a major global health concern, with approximately 10,000 pregnant women dying from malaria-related anaemia each year. The World Health Organization has suggested intermittent preventive treatment with sulphadoxine-pyrimethamine (IPTp-SP) to avert malaria infection in pregnant women in malaria-endemic areas, but this intermittent preventive (IP) treatment is at risk of becoming ineffective due to parasite resistance and the contraindication in HIV-infected women. This paper argues that alternative IP treatments such as dihydroartemisinin-piperaquine (DP) should be explored, alongside the urgent need to investigate antimalarial cycling strategies. Additionally, the cost-effectiveness of IPTp-DP should be evaluated, as well as potential barriers to IP treatment such as medication stockouts, late attendance at antenatal clinics, lack of autonomy and freedom among women, and lack of knowledge about malaria prevention. Health education focusing on malaria prevention should be incorporated into routine antenatal care programmes to improve patient compliance. A comprehensive approach that includes the administration of IPTp-DP alone along with other measures such as insecticide-treated nets and medical education is the key to addressing the devastating effects of malaria infection in pregnant women.

## Introduction

Malaria during pregnancy is a global health crisis, endangering around 25 million pregnant women at risk of malaria infection [1]. Urgent solutions are needed, especially in sub-Saharan Africa, where the prevalence of malaria in pregnant women is highest [2]. Malaria during pregnancy can lead to various adverse outcomes such as maternal anaemia, stillbirth, spontaneous abortion, low birth weight, and neonatal death [3]. The heightened risk and severity in pregnant women stem from a compromised immune system and the vulnerability of the placenta to malaria infection. The World Health Organization (WHO) recommends three primary strategies to combat malaria in pregnant women in endemic areas: insecticide-treated net (ITN) usage, timely diagnosis and treatment, and intermittent preventive treatment with sulphadoxine-pyrimethamine (IPTp-SP) (SP) [2]. Limited healthcare access, medication resistance, HIV coinfection, poor compliance, and lack of knowledge challenge treating malaria in pregnant women in sub-Saharan Africa. Furthermore, malaria parasites in sub-Saharan Africa display resistance to common medications. Some effective IP treatments are unsuitable for pregnant women with HIV coinfection, further complicating IP treatment. Noncompliance can occur due to various barriers and misunderstandings. Overcoming these challenges is crucial as sub-Saharan Africa has the highest prevalence of malaria in pregnant women, with severe consequences for both mother and foetus.

This paper will argue that relying solely on intermittent preventive treatment with SP as prophylaxis for malaria in pregnant women is no longer effective, despite current WHO guidelines. The scientific community must explore the use of dihydroartemisinin-piperaquine (DP), to ensure successful prevention of malaria in pregnant women residing in malaria-endemic areas.

### The declining effectiveness of IPTp-SP

The scientific community has traditionally relied on SP as prophylaxis against malaria in pregnant women, with the WHO recommending monthly administration of SP at every antenatal visit starting in the second trimester of pregnancy [4]. However, SP has waning effectiveness, and a burgeoning concern is the escalating problem of *Plasmodium falciparum* resistance to SP [5]. Furthermore, SP is limited in effectiveness by a significant contraindication in HIV-infected pregnant women who are treated with co-trimoxazole prophylaxis for opportunistic infections [2], leaving ITNs as the primary method of prevention. With 15.9 million women in sub-Saharan Africa infected with HIV, this poses a significant problem [6].

### IPTp-DP

The declining effectiveness of SP has led to the proposal of alternative IP treatments, such as IPTp-DP (DP). Studies have shown that DP is more effective than SP at preventing malaria infection in pregnant women. Olaleye et al. [7] found that ‘IPTp-DP may reduce maternal and placental malaria compared to IPTp-SP, and that monthly DP is more effective than SP in reducing placental malaria’ [7]. While DP is effective in preventing malaria infection in pregnant women, no significant difference has been observed in preventing maternal transmission of malaria to the foetus compared to SP [4]. Despite the high level of resistance to *P. falciparum*, Salman et al. have suggested using DP in combination with SP in HIV-negative patients [8]. The continued use of SP despite its waning effectiveness is due to its positive effects on foetal outcomes in the setting of malaria, particularly significantly improved birth weight (mean difference of 69 g, 95% CI 26–112). Malaria infection during pregnancy is a major risk factor for low foetal birth weight, making DP a promising alternative [4].

### Advantages and disadvantages of IPTp-SP and IPTp-DP

SP and DP are two options for IPT for malaria in pregnant women, each with its own advantages and drawbacks. SP is affordable, widely available, and has been used for a long time. It is familiar to healthcare providers and pregnant women and has a well-established safety profile. However, as antimalarial resistance to SP increases, DP may be a more effective alternative in certain settings. Despite being more expensive and less familiar, DP has a longer half-life, once-daily dosing, and good tolerability. It can also be safely combined with co-trimoxazole prophylaxis for pregnant women with HIV infection. Tailoring the approach to a specific context is crucial for optimising malaria prevention in pregnant women. DP exhibits lower resistance compared to SP due to its combination therapy, which includes dihydroartemisinin, an artemisinin derivative. This combination prevents resistance by rapidly reducing the parasite load and targeting different stages of the malaria cycle. In contrast, SP is more susceptible to resistance due to potential mutations in the targeted enzymes, leading to decreased effectiveness and the emergence of mutant strains [9].

DP is also more effective than SP in malaria-prone areas, but assessing safety and tolerability is crucial. Limited studies comparing DP and SP in pregnant women found that SP caused more side effects such as nausea, dizziness, and headaches [10]. SP also showed potential teratogenicity in the first trimester [11]. In a study involving school-aged children in Uganda, DP > amodiaquine (AQ) + SP > SP in terms of parasitaemia risk at 42 days. DP and AQ+SP significantly improved haemoglobin levels, but AQ+SP had a higher risk of immediate vomiting. Serious toxicities were associated with SP and AQ, including severe cutaneous reactions with SP and neutropenia and hepatotoxicity with AQ. DP was found to be more efficacious, safer, and better tolerated than SP alone and AQ+SP [12].

### Cost-effectiveness of IPTp-DP

The cost-effectiveness of SP versus DP depends on factors like medication cost, malaria incidence/prevalence, and drug resistance. In areas with high antimalarial resistance, DP is more cost-effective due to its higher protective efficacy. A cost-effectiveness analysis using data from trials in Kenya and Uganda compared DP and SP for preventing malaria in pregnant women. DP was found to be more effective but more costly, with an incremental cost-effectiveness ratio (ICER) of $8 per DALY averted compared to three doses of SP and $25 per DALY averted compared to monthly doses of SP [13]. Shifting towards DP is justified in malaria-endemic areas with increasing resistance. By calculating the ICER, we can determine the cost-effectiveness of DP versus SP. For instance, in Nigeria, three doses of SP cost $0.93–$1.20 [14], while three doses of DP prevent 892 DALYs compared to SP’s 534 DALYs for 1000 women [13]. Using the formula ICER = (Cost of DP – Cost of SP with antimalarial resistance)/(DALYs averted by DP – DALYs averted by SP with antimalarial resistance), we can establish the cost of DP required for cost-effectiveness. If DP currently costs $4 for three doses, the ICER is 8.6, indicating an $8.60 cost to prevent one additional DALY when switching from DP to SP.

As the cost of DP decreases and the number of DALYs averted by SP decreases, the ICER decreases too. Lowering the cost of DP relative to SP can increase its cost-effectiveness, even if DP’s effectiveness remains constant. The ICER calculation identifies the threshold at which SP is no longer cost-effective, which varies across regions and may change over time. However, it is important to note that even with a positive ICER, indicating a higher cost to prevent an additional DALY when switching from SP to DP, there may still be a preference for DP for various reasons, for example, perceived benefits, patient preferences, or other considerations beyond cost-effectiveness alone. To further assess cost-effectiveness, we can compare the estimated cost per DALY averted in countries with a low Human Development Index (HDI), which is reported as $998 in some literature [15]. By comparing this value to the cost-effectiveness ratios of DP and SP, we can determine how the use of DP and SP aligns with the cost-effectiveness threshold in these settings. Based on our previous values, both SP and DP have cost-effectiveness below the $998 threshold. This indicates that both treatments are considered cost-effective compared to other healthcare expenditures. However, it is important to note that this analysis assumes a ‘do-nothing’ alternative scenario. In reality, the goal is to reduce the $998 cost per DALY averted, which can be achieved by selecting treatment regimens based on the ICER. This approach informs decision-making and prioritises interventions that allocate resources efficiently to improve health outcomes in low HDI countries [15].

### Antimalarial cycling strategy

There is an argument that utilising DP as part of an antimalarial cycling strategy could serve to delay resistance to SP and maintain its efficacy while simultaneously addressing the escalating costs associated with exclusively employing DP over SP, thus potentially offering a more cost-effective approach. This strategy involves rotating different medications to reduce selective pressure on the parasite [16,17]. Boni et al. even suggest that in theory, the most optimal strategy would be to use all available ACTs in rotation to reduce selection pressure and extend the therapeutic lives of the available drugs [16]. Cycling Rotating DP and SP can lower resistance risk and minimise costs. DP combines dihydroartemisinin, a derivative of artemisinin, with piperaquine. Dihydroartemisinin produces free radicals that damage *P. falciparum* parasites [18], while piperaquine accumulates in the parasite’s food vacuole, disrupting haem detoxification and polymerisation, leading to parasite death [19]. In contrast, SP contains sulphadoxine and pyrimethamine, which inhibit enzymes in the parasite’s folate synthesis pathway. Sulphadoxine competes with dihydropteroate synthase, and pyrimethamine inhibits dihydrofolate reductase [20]. Malawi serves as an illustrative example of the potential effectiveness of antimalarial cycling strategies. In 1993, Malawi transitioned from using chloroquine to SP as a first-line antimalarial IPT. Subsequent studies have demonstrated a significant outcome: the re-emergence of chloroquine-sensitive *P. falciparum*, which now predominates in the region after previously displaying resistance to antimalarial medications [21]. This resurgence signifies the possibility that cycling approaches could enhance the sensitivity of parasites to previously ineffective medications, thus enabling the reintroduction of earlier generations and more cost-effective treatments. It is important to note, however, that cycling extends SP’s lifespan but does not prevent resistance altogether.

## Conclusion

The primary finding of this paper suggests that although DP has demonstrated superior efficacy in malaria prevention when compared to SP, its cost-effectiveness may vary across different settings. There are circumstances where SP remains a viable option. However, in regions characterised by high levels of antimalarial resistance, incorporating DP into an antimalarial cycling strategy may be necessary to maintain efficacy. Moreover, it is crucial to address various barriers to IPTp in general, such as affordability, health education, trust in the public health system, and access to resources. Additionally, there remain unresolved research inquiries regarding DP, including its safety and effectiveness in different populations and settings. Obtaining answers to these questions is vital to support the widespread adoption of DP in IPTp. The WHO Global Malaria Programme coordinates global efforts to control and eliminate malaria based on the ‘Global technical strategy for malaria 2016–2030’. Key strategies include diagnostic testing, treatment, monitoring antimalarial medicines, and managing drug resistance [22]. Ultimately, transitioning from SP to context-specific DP for IPTp will be indispensable in accomplishing the objectives outlined in the ‘Global technical strategy for malaria 2016–2030,’ aiming to reduce the morbidity and mortality associated with malaria infection in pregnant women.

## Paper context

Malaria in pregnant women is a significant global health concern in sub-Saharan Africa due to limited healthcare access, medication resistance, and HIV. This paper compares the efficacy, cost-effectiveness, and resistance of IPTp-SP and IPTp-DP and emphasises the need for a comprehensive approach to reducing morbidity and mortality associated with malaria infection in pregnant women.
